# Movement Restriction and Increased Surveillance as Efficient Measures to Control the Spread of Highly Pathogenic Avian Influenza in Backyard Productive Systems in Central Chile

**DOI:** 10.3389/fvets.2020.00424

**Published:** 2020-07-24

**Authors:** Francisca Di Pillo, Pedro Jimenez-Bluhm, Cecilia Baumberger, Víctor Marambio, Pablo Galdames, Gustavo Monti, Stacey Schultz-Cherry, Christopher Hamilton-West

**Affiliations:** ^1^Nucleo de Investigaciones Aplicadas en Ciencias Veterinarias y Agronómicas, Universidad de Las Américas, Santiago, Chile; ^2^Departamento de Medicina Preventiva, Facultad de Ciencias Veterinarias y Pecuarias, Universidad de Chile, Santiago, Chile; ^3^Instituto de Medicina Preventiva Veterinaria, Facultad de Ciencias Veterinarias, Universidad Austral de Chile, Valdivia, Chile; ^4^Department of Infectious Diseases, St. Jude Children's Research Hospital, Memphis, TN, United States

**Keywords:** highly pathogenic avian influenza, backyard production systems, movement restrictions, disease modeling, surveillance

## Abstract

During the last 5 years there has been an alarming number of reports of highly pathogenic avian influenza worldwide. However, little is known about the status of this disease in South America. Chile has been the only country in South America where an HPAI outbreak was reported. This outbreak occurred in 2002 and was due to an H7N3 HPAI, where the most plausible hypothesis that explained the entrance of the disease to the country, had relation to migratory wild birds. Commercial poultry farms in Chile are highly integrated and have high biosecurity standards. Nevertheless, poultry backyard production systems lack biosecurity measures and are widely distributed. Since 2002 outbreak, avian influenza viruses have been identified in wild birds and different animal species kept in backyard productive systems (BPS) in Chile. The aim of this study was to simulate the possible natural history of HPAI after its introduction to BPS in central Chile and to simulate different intervention strategies. To do so, the North American Animal Disease Spread Model version 3.3 was used. The results showed that a median of 15,930 BPS would be affected if HPAI spread among BPS in central Chile, representing 97.8% of the current amount of BPS existing in study zone. Movement restrictions, pre-emptive destruction, passive surveillance, tracing of infected premises and combinations of the three, where the intervention strategies tested in the simulation model. From all the interventions simulated, movement restrictions together with increasing surveillance (through increasing passive surveillance and good tracing of infected premises) had the biggest effect, reducing the median number of infected BPS in 90.8%. However, more studies are needed to more accurately estimate local contact rates. These results can guide the official veterinary services to consider potential mechanisms to control or prevent an HPAI emergency situation.

## Introduction

Avian influenza (AI) is a disease of global concern divided into two groups depending on its pathogenicity in poultry: (i) highly pathogenic avian influenza (HPAI) causing high mortality (up to 100%) to domestic poultry (1) and (ii) low pathogenic avian influenza (LPAI) which includes viruses causing a milder respiratory disease (2). Wild birds have been identified to be mostly asymptomatic reservoirs of all AI subtypes (3) and they have been proposed as the most likely route of introduction of LPAI viruses into domestic poultry populations (4).

Highly pathogenic avian influenza has affected domestic poultry in 68 countries and territories since 2013, involving 7,060 outbreaks and a high diversity of circulating subtypes. The health and economic impact of these outbreaks has been outstanding, with 57% of all domestic poultry losses reported in Asia, followed by the Americas (24%) and Europe (12%) (5). However, there is little knowledge of AI status in South America (6). In fact, Chile has been the only country where outbreaks of LPAI and HPAI have occurred. In 2002, an H7N3 HPAI virus affected commercial farms in central Chile (7). The origin of the outbreak was associated to migratory wild bird as there was a correspondence between an H7N3 avian influenza virus (AIV) isolated from a Cinnamon Teal (*Anas cyanoptera*) in Bolivia in 2001 and the H7N3 virus isolated in the Chilean 2002 outbreak (8). More recently, an H7N6 LPAI virus was detected in two commercial turkey farms in central Chile, which also had origin in wild birds (9). Importantly, AI reactive antibodies were detected in samples of backyard poultry during active surveillance control activities of this outbreak, and as a result elimination of ELISA positive birds from detected and contiguous households was ordered (10). Additionally, pmdH1N1 and H4N8 have been detected in turkey farms during active surveillance activities carried out by the Chilean official veterinary service (11).

Chilean poultry production is highly integrated at the industrial level, operating with high biosecurity standards (12) and in close cooperation with the official veterinary service. However, in backyard production systems (BPS) the biosecurity implementation and rapid outbreak response activities are very limited and usually absent (12). BPS are usually defined as those productive systems where different animal species, mostly of different ages, are kept in close contact, with poor infrastructure and where the purpose of the production is mainly household consumption (13). Usual production number in poultry BPS in Chile is under 100 birds (12). Birds are usually allowed to roam freely during the day and are confined only during the night, which enable close contact with poultry from neighboring BPS and wild bird species (12, 13) that could act as potential reservoirs of pathogens (12, 14, 15). Therefore, it has been suggested that BPS could play a role in the dissemination of poultry diseases such as HPAI (12, 14, 16, 17). This has been confirmed in recent studies in Chile, where LPAI viruses have been identified in wild birds (18) and domestic poultry kept in BPS (19). On the one hand, Bravo-Vasquez et al. (19), detected influenza A positive matrix gene (rRT-PCR) simultaneously in poultry, swine and geese from BPS, with viral prevalence levels of 27% (95% CI:14–39) in samples from poultry. While in wild birds, three LPAI subtypes (H5N9, H13N2, H13N9) have been detected in gulls in the late 2000's (20). In addition, a recent study in wild birds in central and northern Chile obtained an overall prevalence of 2.8%, isolating 16 viruses, including low pathogenic H5 and H7 strains, making it the largest and most diverse collection of Chilean AIVs to date (18). The same authors also detected an H12 hemagglutinin (HA) sequence from wild birds in one domestic Muscovy duck, indicating a spillover from wild birds into backyard poultry populations (6).

To date, there is uncertainty of how the virus would spread among BPS if an HPAI outbreak occurred at this level in central Chile.

The use of epidemiological modeling has been found to be a useful approach to estimate the possible magnitude of a disease outbreak and the resources that would be necessary for a rapid response and disease control planning (21), while preventing the sustained widespread epidemic among poultry (22), protecting regions from the potentially serious socio-economic consequences of an outbreak (23) and reducing possible human exposures (24).

The aims of this study are to simulate the impact of an outbreak of HPAI at the BPS level in central Chile and to identify variables that could influence the number of affected backyards, in order to design control strategies for possible outbreak within this type of production system.

## Materials and Methods

### Study Area

The study area was 48,186 km^2^ of the central zone of Chile, including three administrative regions: Valparaiso, Metropolitan, and Libertador General Bernardo O'Higgins (LGB O'Higgins). This area compromise ~95% of the commercial poultry production in Chile and 16,289 BPS that keep poultry (25). For this study, BPS that breed poultry were considered the study unit.

### Modeling Framework

The North American Animal Disease Spread Model (NAADSM version 3.3) was used for the simulation of disease spread and control between flocks (26, 27). NAADSM is a stochastic, state transition model framework which incorporates spatial and temporal information to simulate the spread of highly contagious animal diseases. Additionally, the software includes a package that allows modeling the spread of the disease within-herd (WH package), which can be done in a phase prior to entering the data into the NAADSM model. The Within-herd (WH) is a deterministic model (28) used to simulate the dynamics of disease spread and immunity at individual level within a homogeneously mixing population (flock). Detailed information regarding how these parameters were entered into WH and NAADSM and the information sources used for each individual value are presented below.

### Within-Herd (Flock) Model

#### Input and Disease Transmission Parameters

Input parameters in WH included information about animal population demographics and disease state duration and transmission. Four disease states were considered: susceptible, latent, clinically infectious and immune. Each bird could only be in one particular state of the disease at any given time point. The length of time that each bird remained in the latent and clinical disease states were derived from literature review (23). An immune period of 1,000 days was used so that the model did not consider as susceptible, birds that had already been infected. Parameters values used in the WH model are shown in Tables 1, 2. The time step considered was a day and 1,000 iterations were run. Outcomes produced by the WH model were then used to develop flock-level parameters for NAADSM.

**Table 1:** Population data included in the WH model for the dissemination of HPAI virus in BPS.

**Variable**	**Value**	**Distribution/Parameter value**	**References**
Population size	44 birds	Loglogistic (−0.12; 31.13; 2.27)	(13)
Initially latent individuals	1 animal	Point (1.00)	User defined
Initially clinical animals	0 animals	Point (0.00)	User defined
Adequate exposures per time step	1.7	Poisson (1.7)	(29)

**Table 2:** Values of disease state duration and mortality parameters for individual birds in BPS used in the WH model.

**Parameter description**	**Distribution/Parameter value**	**References**
**DISEASE**		
Latent period	Exp (1) (CI: 0.05; 3.00)	(23)
Clinical period	Gaussian (4.00; 1.00) (CI: 2.5; 5.8)	(23)
Immune period	Point (1,000)	User defined
**MORTALITY**		
Probability that disease will result in death	Point (0.9)	(29)
Probability of non-disease death	Point (0.00005)	(29)

### Between-Flock Transmission Model

#### Input Parameters

Input parameters included information about animal populations, disease presentation, disease transmission between flocks, disease detection and surveillance and disease control. Each BPS was considered to be a single unit. Each unit in NAADSM is characterized by its (i) production type, (ii) herd size (number of birds in the flock), and its (iii) spatial-location (latitude and longitude coordinates).

- Production type

A production type is a collection of herds with similar virus transmission probabilities, disease presentation, disease detection probabilities, and control strategies (28). For this study, the only production type considered was BPS.

- Flock size

A set of 16,289 different flock sizes was created and generated from a database of 384 known BPS obtained from previous studies in central Chile (13). The distribution of the BPS flock size was described using @Risk 7.5 Palisade software (Ithaca, New York). The distribution was fitted for the 384 BPS sizes and values for all flock sizes to be used were generated from the fitted distribution with a simulation of 16,289 iterations.

- Geolocation

**Figure 1:**
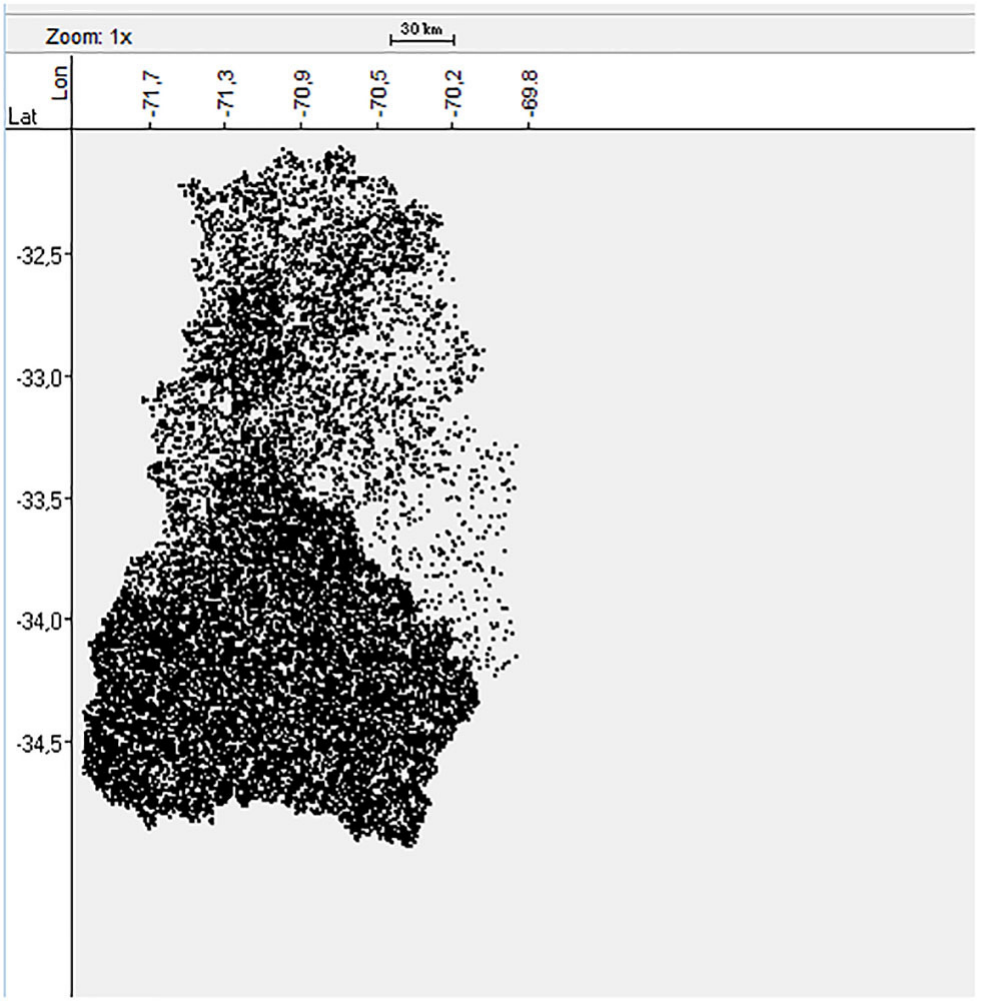
Spatial distribution of the 16,289 BPS in the central Zone of Chile.

The forestry, agricultural and livestock census published in 2007 provided the information about the number of BPS located in each province of the central zone of Chile (25). However, the exact geolocation of each BPS was not known. Therefore, a dataset of 16,289 random geolocations was generated and stratified by province (Figure 1) according to the census information using the Surface Tool in ArcGIS-10 software (Esri, California, USA). This methodology was previously validated in Chile by Alegria-Moran et al. (30) indicating that the approach followed a realistic spatial distribution.

#### Disease Manifestation

Four disease states were used: susceptible, latent, clinically infectious and immune. The length of time that each flock remained in the latent and clinical disease states were derived from literature review (23). As described for the WH model, an immune period of 1,000 days was used so that the model did not consider as susceptible a BPS that had already been infected.

#### Between-Flock Transmission

Between-flock virus transmission considered direct contact, indirect contact and local-area spread between infected and susceptible flocks. Table 3 shows the parameters and values used for the simulation. Indirect contact considered movement of people, vehicles, materials and animal products between flocks. Direct contact spread considered the movement of one or more birds from one flock to another. To represent non-directional local-area spread (i.e., disease spread that cannot be well-characterized or traced such as spread by insects, pests, lapses in biosecurity, and local airborne transmission) the airborne spread function in *NAADSM* was used.

**Table 3:** Values and sources of disease transmission parameters for the between flock simulation.

**Disease**	**Distribution/Parameter value**	**References**
Latent period	Gamma (1.3; 0.8)	(23)
Infectious clinical period	Logistic (14.88; 1.7)	WH model outcome
Immune period	Point (1,000)	User defined
Within herd prevalence	Relational function	WH model outcome
**DIRECT CONTACT SPREAD**		
Mean baseline contact rate^*^	0.4098	2015–2017 Database
Probability of infection transfer	Determined by WH prevalence	WH model outcome
Distance distribution of recipient units	BetaPERT (0, 3, 10)	User defined
**INDIRECT CONTACT SPREAD**		
Mean baseline contact rate^*^	0.5529	2015–2017 Database
Probability of infection transfer	0.5	(29)
Distance distribution of recipient units	BetaPERT (0, 3, 20)	User defined
**AIRBORNE SPREAD**		
Probability of spread/day, at 1 km	0.05	(29)
Start, End	0.360	

* *Recipient units/units/day*.

The parameters used to define virus transmission by contact were: mean baseline contact rate, probability of infection transfer and distance distribution of recipient flocks (26). Latent flocks were assumed to be able to spread virus only by direct contact. Infectious clinical flocks could spread the virus to other flocks via direct contact, indirect contact and local-area spread. Flocks in the immune state were not able to spread the virus or become infected.

##### Virus transmission by direct contact

Direct contact involved birds in a source BPS coming into contact with birds in a recipient BPS. The direct contact rate was the average daily number of shipment of birds which could introduce the virus into new flocks. This value was derived from previous studies in Chile carried out during 2015–2017 where a semi-structured survey was applied to 384 BPS in central Chile (13). The data collected in the database included information that allowed the characterization of direct and indirect contact rates between BPS. It also allowed the collection of information regarding the owners' ability to recognize when their birds were sick, as well as information regarding the actions they take against large bird mortalities. Those data described that 40.9% of the owners reported that at least 1 bird from their neighbors entered their BPS and contacted their birds daily. Additionally, 35.6% of the owners indicated buying birds for replacement an average of 0.83 times a year. As only one field exists in NAADSM to describe direct and indirect contact rates, the estimated daily frequencies of each type of direct contact were added to generate an overall average daily number of direct contacts (Table 4).

**Table 4:** Sources of direct contact from backyard to backyard poultry production.

**Direct contact**	**Formulae**	**Frequency**	**Per week**	**Per day**
Neighbor's birds	1.0 × 0.409	1.0/day	-	0.409
Birds replacement	0.83 × 0.36	0.3/year	0.0058	0.0008
Total	-	-	-	0.4098

The probability of virus transmission was determined by the prevalence of infectiousness in the infected BPS on the day that the contact occurs. Estimates of the median daily prevalence of infectiousness for an infected backyard flock were produced using the WH model outcomes. The distance distribution of recipient BPS was assumed to have a BetaPert distribution with a minimum of 0, a mode of 3 and a maximum of 10 kilometers.

##### Virus transmission by indirect contact

The contact rate for indirect contacts was the average daily number of movements of people, vehicles, equipment, materials or animal products from a source flock (26) to a recipient flock. The estimated daily frequencies of each type of indirect contact were added to generate an overall average daily number of indirect contacts. The sources of indirect contact rates and their values were derived from the 2015–2017 mentioned database and are described in Table 5. The distance distribution of recipient flocks was assumed to have a BetaPert distribution with a minimum of 0, a mode of 3, and a maximum of 20 km. These distances were considered from Di Pillo et al. (13) results that described BPS poultry owners movement to access markets.

**Table 5:** Sources of indirect contact from backyard to backyard poultry production.

**Indirect contact**	**Frequency**	**Per week**	**Per day**
Neighbors visits	3.8/weeks	3.8	0.54
Bird food purchase	0.33/months	0.0825	0.0118
Veterinary care	0.128/years	0.0025	0.0004
Embryonated eggs exchange	0.26/years	0.005	0.0007
Total	-	3.89	0.5529

##### Virus transmission by local-area spread

The probability of infection by local-area spread was considered a relational function depending on the prevalence of infectiousness in an infected flock and the distance between a source and a recipient flock. The probability of infection by local-area spread decreased as the distance between flocks increased and the probability of virus transfer declined exponentially from the source flock (29).

#### Disease Detection and Control Parameters

In NAADSM, the overall chance that an infected flock will be detected depended on two probabilities, the probability that clinical signs are observed in an infected flock, and the probability that a flock is reported once clinical signs have been observed (26). Values for each probability were derived from (13) and 2015–2017 database, where 80% of poultry owners declared to recognize when their birds were sick. While the probability of reporting flocks with clinical signs derived from the sum of two components. One point eight per cent of producers declared reporting mortalities to the official veterinary service. While 10% of producers reported having visits from the official veterinary service. Thus, a report of 11.8% (0.12) was assumed.

#### Control and Prevention Measures

The baseline scenario represented the natural history of the disease (no intervention measures are taken) once it entered a BPS. The effect of different strategies on the total number of infected BPS and outbreak duration against baseline scenario were evaluated. These interventions included (i) Movement restrictions, (ii) tracing of infected BPS, (iii) depopulation and pre-emptive destruction and, (iv) increasing passive surveillance (probability of reporting infected BPS). The effect of different combinations of strategies were evaluated.

Movement restrictions considered the reduction in the number of contacts between BPS, thereby reducing the possibility for disease spread. This restriction on movement was considered in two ways; (i) restriction from the beginning of the epidemic, to reflect the total confinement of birds as a preventive measure, and (ii) restriction from day 3 after the detection of disease, to reflect the effect of prohibiting the movement of birds and people as a control measure once the epidemic has started. Movement restrictions of 50% and 90% of the baseline contact rates were simulated.

Global Tracing consisted of the process of identifying units (BPS) at high risk for disease based on contact with detected units (26). The critical period considered for direct and indirect tracing was 14 days. This is the period of time prior to detection of the origin unit of the trace, for which contacts should be investigated (26). The probability of trace success for direct and indirect contacts were derived from expert opinion and were 0.8 and 0.5, respectively. Herd exams were also included and a multiplier of 1.5 for trace-forward contacts and 2.0 for trace-back contacts were assumed. Parameters for herd exams are multipliers that describes how much more likely a trained observer is to detect clinical signs compared to more passive observers (26). Tracing parameters also included diagnostic testing of traced BPS. The sensibility and specificity used were 95.4 and 99.7% (19). A delay in obtaining tests results was considered to have a BetaPert distribution of 0, 3, and 7 days.

Depopulation considered the destruction of infected and detected BPS. While pre-emptive culling considered culling of potentially uninfected BPS in a ring radius of 3 km around a detected BPS. The delay in implementing the stamping out programs was simulated using two options, a 7 days delay and a quicker response of 2 days delay. Once the stamping out program started, it considered two different amounts of BPS slaughtered daily. The first and more conservative option considered the destruction of 10 BPS daily during the first week, with an increase in capacity to the destruction of 20 BPS per day since day 7 after the destruction program started. The second option considered the destruction of 100 BPS daily since day 2 of detection.

Improvement of passive surveillance was simulated by increasing the probability of reporting flocks with clinical signs to 0.9 since day 1 of disease first detection in any unit.

#### Assumptions

The model developed laid on the following assumptions: (i) once a BPS become infected, it left the poultry business, thus no repopulation of birds existed, (ii) it was a homogeneously mixed population, (iii) there was no transmission of the virus between BPS and commercial farms, or vice versa, due to the high biosecurity standards applied in the latter in Chile and (iv) it was assumed that all the birds present were domestic chickens. Although usually different species of birds coexist in BPS, previous studies (13) have described that 87% of the population of birds in these systems correspond to domestic chickens. In this way, the parameters used in the model correspond to those described for domestic chickens.

#### Model Outcomes

Each simulated outbreak started with a single, randomly selected latently infected BPS, in a totally susceptible population of BPS. For each of the scenarios, 1,000 iterations were run. Each iteration ran until the end of outbreak which was defined as the moment when there were no more latent or clinically infectious BPS left, and when all destruction activities were completed. The outcomes of interest were the total number of infected BPS (BPS infected by any path), time (days) to first detection of infected BPS in the population, the total number of detected and infected BPS and the outbreak duration. For each output, the summary statistics calculated were the median, 5th percentile and 95th percentile.

## Results

### Input Parameters

#### BPS Poultry Population Demographics

Of the 16,289 BPS, the total number of birds was 713,665, distributed in flocks ranging from 2 to 300 birds, with a mean of 44 (SD = 39.2) birds/flock and a median of 30 domestic chickens (IQR: 20–50).

### Simulation Results

When simulating the basal scenario, the great majority of virus transmission was due to indirect contact, accounting for 99.3% of the infected BPS. The median duration of the basal scenario outbreak was 314 days (p5 = 247; p95 = 372), with a median number of infected BPS of 15,930 (p5 = 15,889; p95 = 15,961), meaning that 97.8% of the total backyard population got infected when no intervention measures were applied. Disease detection occurred in 890 of 1,000 iterations, being the median time to first detection 97 days (p5 = 50; p95 = 209). The median total number of birds infected were 701,746 (p5 = 699,846; p95 = 703,306). The model outcomes are shown in Table 6.

**Table 6:** Baseline model outcome generated from 1,000 stochastic iterations of the model of HPAI in BPS in central Chile.

**Baseline model outcome**	**Median**	**P5**	**P95**
Total number of infected flocks	15,930	15,889	15,961
Number of flocks infected by direct contact	58	45	70
Number of flocks infected by indirect contact	15,826	15,783	15,861
Number of flocks infected by local-area spread	46	33	56
Total number of birds in infected flocks	701,746	699,846	703,306
Time to first detection (days)	97	50	209
Total number of detected infected flocks	2	0	5
Outbreak duration (days)	314	247	372

### Control and Prevention Measures

The greatest impact on the median number of infected BPS and outbreak duration was movement restriction (Table 7). When movement restriction was set to 90% as a preventive measure (birds totally confined since day 1), there were not infected BPS and the outbreak did not launch. On the other hand, implementing movement restrictions once the epidemic had already started (since day 3 of the first detected infection of a BPS), the outbreak did launch, but it was controlled within 122 days and the amount of infected BPS decreased in 91%. However, in order to see a greater effect, it was necessary to carry movement restrictions together with other intervention strategies, such as increasing the probability that owners reported when their birds became infected and to perform tracing of the disease. When passive surveillance (probability or reporting infected BPS) was not included, the infected BPS decreased in 69%.

**Table 7:** Effect of control and prevention measures over the median number of infected BPS, outbreak duration, time to first detection, and number of detected infected BPS.

**Intervention strategy implemented**	**Median number of BPS infected (5th−95th percentile)**	**Median outbreak duration (days) (5th−95th percentile)**	**Median time to first detection (days) (5th−95th percentile)**	**Median total number of detected infected flocks (5th−95th percentile)**
Basal model	15,930 (15,889–15,962)	314 (247–372)	97 (50–209)	2 (0–5)
Movement restriction 90% (day 1) + Increased report 90% + Tracing	0 (0–5)	19 (12–47)	-	0
Movement restriction 90% (day 1) + Increased report 90% + Tracing + Depopulation 10 BPS daily (since day 7)	0 (0–0)	17 (11–22)	-	0
Movement restriction 90% (day 3) + Increased report 90% + Tracing	1,463 (238–4,799)	122 (80–160)	57 (31–98)	468 (1–1,038)
Movement restriction 90% (day 3) + Increased report 40% + Tracing	2,688 (163–8,971)	138 (82–210)	75 (36–138)	469 (2–977)
Movement restriction 90% (day 3) + Tracing	4,904 (671–15,889)	166 (103–297)	101 (52–225)	483 (0–799)
Movement restriction 50% (day 3) + Increased report 90% + Tracing + Depopulation 100 BPS daily (since day 2)	11,665 (10,447–12,674)	564 (432–666)	53 (31–93)	1,396 (344–3,773)
Movement restriction 50% (day 3) + Increased report 90% + Tracing + Depopulation 300 BPS daily (since day 3)	11,749 (10,676–12,598)	531 (376–679)	56 (32–94)	1,421 (661–2,141)
Movement restriction 50% (day 1) + Increased report 90%	12,783 (0–12,959)	569 (15–727)	102 (30–176)	12 (0–20)
Movement restriction 50% (day 1) + Increased report 90% + Tracing + Depopulation 10 BPS (since day 7)	12,966 (0–13,465)	682 (15–828)	104 (58–200)	4,139 (0–5,608)
Increased report 90% + Tracing + Depopulation 100 BPS daily (since day 3)	14,789 (11,211–15,997)	299 (232–374)	58 (30–94)	12,076 (7,122–14,025)
Increased report 50% + Tracing	15,922 (15,876–15,953)	310 (238–362)	68 (31–115)	13,074 (9,247–14,024)
Increased report 50% + Tracing + Depopulation 10 BPS daily (since day 7)	15,927 (15,855–15,964)	852 (674–879)	63 (31–113)	13,235 (8,900–13,932)
Increased report 90% + Tracing + Depopulation 10 BPS daily (since day 7)	15,921 (15,028–15.960)	861 (573–879)	53 (27–80)	13,712 (7,677–13,964)

**Figure 2:**
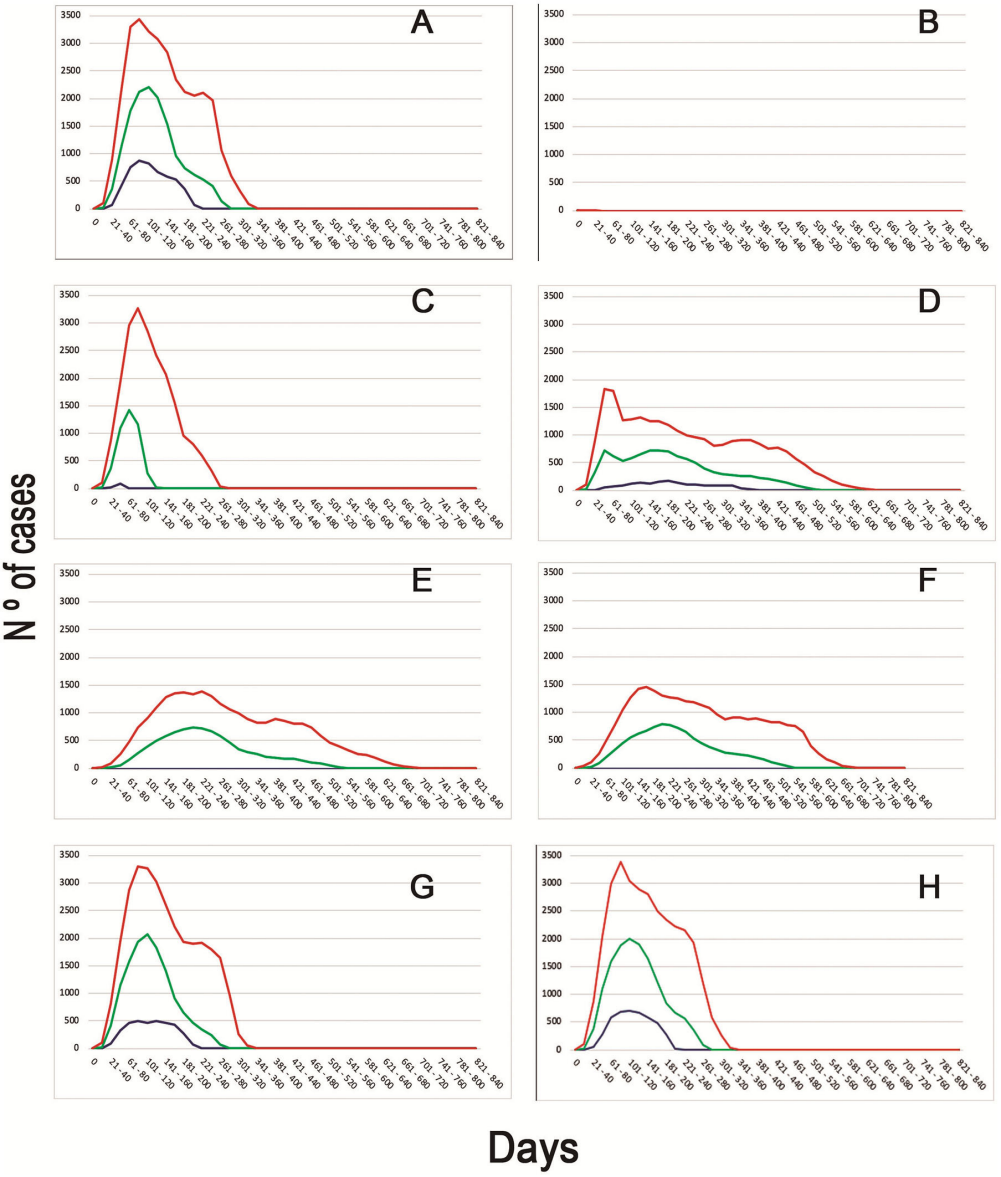
Epidemic curves of the different scenarios simulated. Different colors represent the daily cases of infected BPS (Blue−5th percentile; Green—Median; Red−95th percentile). Letters represent the epidemic curves for the different scenarios **(A)** Basal model with no intervention strategies. **(B)** Movement restriction of 90% (since day 1) + Increased report to 90% + Tracing. **(C)** Movement restriction of 90% (since day 3) + Tracing. **(D)** Movement restriction of 50% (since day 3) + Increased report to 90% + Tracing + Depopulation of 100 BPS daily since day 2. **(E)** Movement restriction of 50% (since day 1) + Increased report to 90%. **(F)** Movement restriction of 50% (since day 1) + Increased report to 90% + Tracing + Depopulation of 10 BPS since day 7. **(G)** Increased Report to 90% + Tracing + Depopulation of 100 BPS daily (since day 3). **(H)** Increased report to 50% + Tracing.

When evaluating the median time to first detection, increasing passive surveillance had the greatest impact, displaying a reduction from 97 days to only 53 days. This strategy had also the greatest impact on median total number of detection of infected flocks, increasing this number to 13,712 in comparison to the basal scenario, when the median total number of detection of infected flocks were just two. The epidemic curves generated from the different scenarios can be observed in Figure 2.

## Discussion

The use of epidemiological modeling of infectious diseases are a useful approach to estimate the possible magnitudes of a disease outbreak and the resources that would be needed to generate a rapid response, but for the models to be applicable to different contexts, it is important that they can consider data from the local susceptible populations in which the model is being applied. In this study, we managed to gather information that describes contact rates between BPS in central Chile, which means that our results can guide local work, in situations of epidemiological calm, in order to respond better to prevent and respond to health emergencies.

The most important finding of this study was that the best control strategy to be used to control an outbreak of HPAI in BPS situations is to apply movement restrictions. This is consistent with that described by several authors, who point out that isolating individuals is one of the oldest, however most effective strategies, to control infectious diseases (31).

This strategy had the biggest effect when implemented in both situations, as a preventive measure confining birds before the outbreak started, and as a control measure once the outbreak started. Improving biosecurity measures at BPS level by building chicken coops could decrease both direct and indirect contact rates. Biosecurity is the first line of defense against an introduction of AI and probably the only defense when preventive/prophylactic vaccination of flocks at risk is excluded (32), as it is in the case of Chile, where vaccination against avian influenza is prohibited. Depending on the circumstances, biosecurity might be defined as biocontainment which is the prevention of the virus of exiting the infected unit; or as bioexclusion, which refers to the prevention of virus introduction into a disease-free unit (33). Naturally, bioexclusion is not easy to achieve in free-range or mixed system. This difficulty has previously been explained due to the free access of wild birds to these systems, which may be carrying the virus (32). Other reason that explains the difficulty of bioexclusion in the case of BPS, is the free movement of domestic birds between different productive units (34). Because of these, poultry farmer organizations in the Netherlands are suggesting keeping poultry inside (confined in chicken coops with roofs) during the wild-bird migration period in spring (32). The construction of chicken coops in BPS would keep the birds confined, reducing both the direct contact rate and the indirect contact rate. The latter, by limiting the amount of feces available in the environment, which can be transferred from one BPS to another through fomites or through movement of people that could carry the virus in their shoes or vehicles (35). In fact, when this scenario was simulated, represented by movement restrictions in 90% of the BPS since day 1, the outbreak did not launch.

A previous study in Minnesota described that free-range and semi-confinement have been the introduction points for LPAI viruses into commercial flocks. In addition to lack of confinement, small flocks of domestic waterfowl, such as ducks and geese raised outdoors are also a possible route of introduction of the virus, particularly if they are reared together with other species of domestic poultry under common handling conditions (32), as it is in the case of BPS in Chile, where most households raise different animal species altogether (chickens, ducks and geese). Furthermore, trading and exchanging live birds may perpetuate the infection and the spread to other farms. In Chile, it has been described that in 74% of BPS households, eggs and poultry are usually given away to relatives/friends. In addition, the exchange of embryonated eggs to improve the flocks productive yield is also a common practice (13). These activities are of crucial importance when considering that transmission of AIV may occur by nearly anything contaminated with fecal material (32), reinforcing the importance of restricting birds and bird's products movement.

Although the local contact rates for Chile were estimated from previous studies in the country, there are still gaps in the information regarding the effect of the contact of domestic birds with wild birds that can carry the virus. Sequence analysis of viruses isolated in an Italian wild bird survey identified that the H7 gene showed a 99.3% homology at the nucleotide level between the isolates from the backyard flocks and the isolates obtained from wild birds (36). As mentioned before, these types of events, have also been described in Chile by Jimenez-Bluhm et al. (6) where the risk and evidence of spillover of an H12 virus from wild birds to BPS is described.

On the other hand, improving passive surveillance by increasing the probability of poultry owners of reporting infected BPS is a key element. The most effective control strategy once the outbreak started, was the combination of rapid bird confinement once the first infected BPS was detected. If the probability of reporting is low, infected BPS are not detected, and implementing any other intervention measure (without increasing passive surveillance) would have virtually no effect in controlling the spread of the disease. In fact, only by increasing passive surveillance, the median time to first detection decreased by 45% of the baseline, which is a known necessary step for ensuring a rapid response in the face of a sanitary emergency (37).

Because BPS in Chile are generally found in remote areas (13), poultry owners are the first link in the passive surveillance chain for an early warning system. So, educating poultry owners on HPAI would become therefore a crucial prevention strategy. The Italian and Dutch experiences have shown that a delayed detection of an HPAI epidemic in a high-density poultry area, makes it more difficult to control the disease (38, 39). The consequence of not reporting or overlooking AI suspect cases because of low specificity of clinical signs, lack of knowledge about the disease or because people are used to being in a disease-free country, would allow the virus to have a longer period for its dissemination. A longer high-risk period increases the risk of spread of infection to other BPS, a fact that could seriously hinder the efforts of eradicating AI after its introduction into a country free of the disease (32).

In addition, training personnel from official veterinary services to trace contacts from an infected backyard has an important effect on the probability of detecting infected BPS. However, measures such as depopulation, had a low impact on the simulated scenarios. Nevertheless, simulating the responsiveness of the official veterinary service can be a difficult task when official data are not available. However, the previous simulated measures (confinement and increased passive surveillance) are preventive measures easy to implement independently from the official veterinary service.

The base scenario simulated in this study, pointed out that 97.8% of the total backyard population would get infected with HPAI if no intervention measures were applied. With this background, it is necessary to improve the preparation of BPS. This preparation could be achieved by improving biosecurity together with an education campaign, with the aim of having a better passive surveillance and thus decreasing the probability of transmission. On the contrary, the commercial poultry industry in Chile constantly invests in improving its levels of biosecurity, attain advances in the development of geographic compartments and constantly performs active and passive surveillance, activities that should also be carried out for BPS.

These results would probably be refined if future studies deepen the estimation of contact rates and parameters for the context of Chile. Because of this, the model established for this study is not really intended to forecast the outcomes of an outbreak, but to be used in advance of an outbreak for decision support, planning and preparation.

## Data Availability Statement

The datasets generated for this study are available on request to the corresponding author.

## Author Contributions

CH-W, SS-C, and FD: conceptualization. FD, CH-W, PG, CB, VM, GM, and CH-W: methodology. FD, CH-W, PJ-B, PG, CB, VM, GM, and CH-W: writing-original draft. CH-W, SS-C, and FD: resources. All authors contributed to the article and approved the submitted version.

## Conflict of Interest

The authors declare that the research was conducted in the absence of any commercial or financial relationships that could be construed as a potential conflict of interest.
